# Efficacy and safety of intradetrusor onabotulinumtoxinA injection for managing paediatric non-neurogenic overactive bladder: A prospective case-series study

**DOI:** 10.1080/2090598X.2019.1600993

**Published:** 2019-04-24

**Authors:** Amr S. El-Dakhakhny, Tarek M. El-Karamany, Mohamed El-Atrebi, Tarek Gharib

**Affiliations:** a Department of Urology, Faculty of Medicine, Benha University, Benha, Egypt; b Department of Urology, El-Sahel Teaching Hospital, Cairo, Egypt

**Keywords:** Overactive bladder, intradetrusor botox injection, anti-cholinergic therapy, subjective scoring, urodynamic studies

## Abstract

**Objectives**: To evaluate the outcome of intradetrusor onabotulinumtoxinA (Botox®; Allergan Inc., Dublin, Ireland) (IDB) injection in children and adolescents with non-neurogenic overactive bladder (OAB) refractory or resistant to treatment.

**Patients and Methods**: In all, 91 patients underwent evaluation using subjective scores and urodynamic studies (UDS), including determination of maximum bladder capacity (MBC) and evaluating the capacity deficit vs the expected bladder capacity (EBC), and uroflowmetry determination of voided volume, maximum urinary flow rate (Q_max_) and post-void residual urine volume (PVR). All patients received oxybutynin (0.3–0.5 mg/kg/day) for 3 months and re-evaluated patients who developed drug intolerability, persistence or recurrence of OAB received 100 U IDB injection using 20 injection sites, with trigone and sphincter sparing. All patients were re-evaluated 3-monthly for subjective scoring and at the end of the 12-month follow-up with UDS.

**Results**: In all, 43 patients underwent IDB injection and at the end of the 12-month follow-up the success rate for IDB injection was 90.7%. All patients showed progressively decreasing scores compared to baseline scores. At the 12-month follow-up, MBC, voided volume, and Q_max_ were significantly higher, whilst capacity deficit and PVR were significantly lower than baseline measures. The frequency of patients satisfied with the outcome of IDB was high.

**Conclusion**: For children with OAB refractory or resistant to biofeedback therapy, anti-cholinergic drugs must be tried first with IDB reserved for cases who fail to respond, are intolerant or recur after medical treatment. IDB using 100 U Botox, at 20 injection sites with trigone and sphincter sparing, is successful with a high satisfaction rate and free of postoperative problems.

**Abbreviations**: EBC: expected bladder capacity; IDB: intradetrusor onabotulinumtoxinA; MBC: maximum bladder capacity; OAB: overactive bladder; OABSS: Overactive Bladder Symptom Score; PPBC: Patient Perception of Bladder Condition; PVR: post-void residual urine volume; TENS: transcutaneous electrical nerve stimulation; Q_max_: maximum urinary flow rate; UDS: urodynamic studies; UI: urinary incontinence

## Introduction

Non-neurogenic lower urinary tract dysfunction in the paediatric population is very common and is one of the important underlying causes of LUTS, UTI and VUR in affected children [1]. Bladder overactivity can be defined as the presence of voiding urgency, associated with increased daytime frequency and nocturia, with or without urinary incontinence (UI), in the absence of UTI or other obvious pathology [2].

Proper management of children with bladder overactivity depends on detailed history taking; validated questionnaire on voiding, voiding diary, urine analysis, ultrasonography, uroflowmetry and post-void residual urine volume (PVR) measurement, but invasive urodynamic studies (UDS) should be reserved for when standard treatment has failed [3]. Management includes the use of combined physiotherapeutic methods aiming to regulate the act of urination through normalising bladder muscle tone, eliminating sphincter insufficiency, improving circulation, and accelerating the maturation of the neuromuscular apparatus of pelvic organs [4].

Pelvic floor biofeedback training should be considered the initial treatment option in patients with non-neurogenic overactive bladder (OAB), as it is an effective treatment modality in children with treatment refractory OAB and dysfunctional voiding [5], or can be used as a supplementary to standard urotherapy [1].

Pharmacotherapy for OAB should have a better chance of curing various problems and improving self-esteem and quality of life in children with hyperactive bladder [1]. Oxybutynin, an M-cholinoblocker, is the ‘gold standard’ for the pharmacotherapy of childhood bladder dysfunction [4]. Transcutaneous electrical nerve stimulation (TENS) is another safe and well-tolerated therapeutic modality for children with OAB refractory to pharmacotherapy [6]. Urodynamic improvements support the efficacy of TENS for OAB management in children [7].

Botulinum toxin is a purified form of the neurotoxin from *Clostridium botulinum* bacteria and has been used in medicine for many years [8]. OnabotulinumtoxinA (Botox®; Allergan Inc., Dublin, Ireland) is a type A neurotoxin derived from *Clostridium botulinum* that is approved as a treatment for UI in patients with neurogenic detrusor overactivity secondary to multiple sclerosis or sub-cervical spinal cord injury who are not adequately treated by antimuscarinics [9]. Intradetrusor Botox (IDB) injection is an effective option for managing patients with neurogenic detrusor overactivity who do not respond to or tolerate oral pharmacological agents [10] and patients whose symptoms are refractory to conventional therapy [11].

In the present study, we evaluated the outcome of IDB injection as a therapeutic modality for children and adolescents with OAB refractory or resistant to treatment.

## Patients and methods

The protocol of this prospective clinical trial was approved by the Local Ethics Committee and parents of enrolled patients signed a written fully-informed consent to participate in the study and receive the assigned lines of management. All children and adolescents aged 5–16 years, with a past history of dryness and recurrence of bed wetting or day wetting later on, and did not improve after 6 months of strict bladder retraining, were eligible for evaluation. Only patients having OAB as diagnosed by UDS were included in the study. Exclusion criteria included: the presence of UTIs both current infections, evidenced by urine analysis and culture, or within the last 2 months; presence of abnormalities of urinary tract or nervous system, and any known genetic or craniofacial syndromes; systemic diseases such as diabetes mellitus, cystic fibrosis; and obstructive sleep apnoea.

### Diagnostic protocol

1. Subjective evaluation: All patients underwent evaluation using:

- Overactive Bladder Symptom Score (OABSS), which assesses four symptoms: daytime frequency (score: 0–2), night-time frequency (score: 0–3), urgency (score: 0–5), and urgency UI (score: 0–5), scored for a sum score ranging between 0 and 15 and with a sum score of >8 suggestive of OAB [12].
- The Patient Perception of Bladder Condition (PPBC) scale: a 6-point scale about the patient’s views of his/her bladder problems and ranging from 1: Not at all, 2: Some very minor, 3: Some minor, 4: some moderate, 5: severe, 6: many severe problems [13].

2. Objective evaluation:

- Complete history taking including history of recurrent wetness after dryness, response to previous therapies and history of corrections of urinary outflow congenital anomalies.
- Complete physical examination to assure exclusion criteria, with special reference to otorhinological evaluation.
- Laboratory investigations including urine analysis and culture, fasting blood glucose levels and kidney function tests.
- Radiological evaluation to exclude congenital anomalies, stones and bladder anomalies.
- UDS using urodynamic equipment (Aymed DYNO Urodynamics, Istanbul, Turkey) including:

- Determination of maximum bladder capacity (MBC) and evaluating the capacity deficit in relation to expected bladder capacity (EBC) calculated for age according to the equation: 30 + [age in years × 30] mL [14].
- Uroflowmetry determination of voided volume, maximum urinary flow rate (Q_max_), and PVR.

### Management protocol

All patients with OAB received oxybutynin at a total dose of 0.3–0.5 mg/kg/day divided into three doses for 3 months. The dose was increased until resolution of symptoms or appearance of intolerable side-effects.

- Patients with persistent UI or had unacceptable trial outcomes or developed significant side-effects with oxybutynin underwent UDS 2 weeks after all anti-cholinergic medications were discontinued and the presence of detrusor contractions during the filling phase (rise of >15 cmH_2_O above baseline) were confirmed and underwent IDB injection.
- Patients showed improved continence and were free or developed tolerable side-effects with oxybutynin continued on the same therapeutic regimen and were followed up 3-monthly for 12-months using both the OABSS and PPBC score, and patients who developed recurrent manifestations at any time during follow-up underwent IDB.

### IDB injection procedure

**Figure 1.**
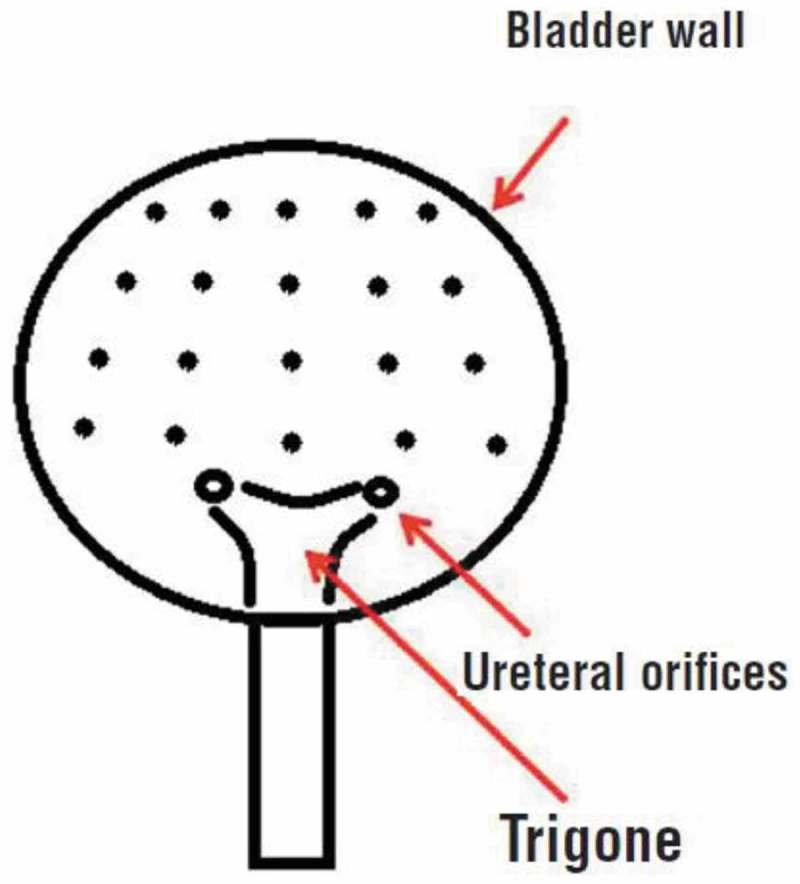
Diagrammatic representation of the sites of IDB injection.

Patients received i.v. anaesthesia and preoperative i.v. broad-spectrum antibiotic. Then, all patients underwent a trigone-sparing procedure with IDB injection at 20 sites, as shown in Figure 1. Botox 100 U was reconstituted with 10 mL saline (0.9%) for a concentration of 10 U/mL. Cystoscopy was performed with an 8-F rigid cystoscope in lithotomy position and after filling the bladder with 100 mL irrigation fluid, IDB injections were performed using a 27-G disposable needle. The depth of injection in the detrusor was ~2 mm, as estimated by the insertion of half of the 4-mm needle. The bladder neck was excluded to avoid outflow obstruction.

### Follow-up

1. All patients received urinary antiseptics and broad-spectrum antimicrobial therapy for 5 days.
2. Urine analysis and urine culture were requested at 2 weeks after IDB injection.
3. The PVR was assessed by the urethral catheter and if it was <15 mL, patients were allowed to continue anti-cholinergic medications at half of the pre-injection dose.
4. The frequency of IDB injection-related complications.
5. Patients were re-evaluated at 3-monthly intervals after IDB injection using the OABSS and PPBC score.

### Study outcome

1. Duration of treatment efficacy was defined as the duration since the start until recurrence of manifestations or the end of the 12-month follow-up.
2. At the end of the 12-month follow-up, patients underwent re-evaluation of MBC and its deficit in relation to the EBC, and of uroflowmetry results. The outcome of the applied therapeutic modality was graded as treatment success (TS) or treatment failure (TF) according to the 12-month objective findings.
3. Patients and/or parents satisfaction of the outcome was graded as: ‘very satisfactory’, ‘satisfactory’, ‘good’, ‘unsatisfactory’, or ‘distressed’.
4. All patients, who developed recurrent OAB manifestations were asked if they wish to undergo another setting of IDB injection or not and this was considered as another form of success.

### Statistical analysis

Obtained data were presented as mean (SD), minimum and maximum values and numbers. Results were analysed using the Student’s *t*-test and chi-squared test. Statistical analysis was conducted using the Statistical Package for the Social Sciences (SPSS®), version 23 (2015), for Windows (SPSS Inc., IBM Corp., Armonk, NY, USA). A *P* < 0.05 was considered statistically significant.

## Results

**Figure 2.**
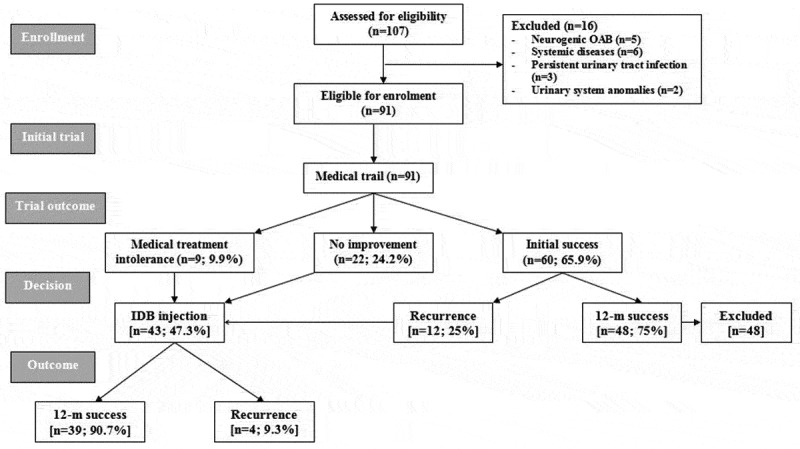
Consolidated Standards of Reporting Trials (CONSORT) flow chart.

The study included 107 patients of which 16 were excluded. The remaining 91 patients comprised 33 boys and 58 girls, with a median (interquartile range) age of 9 (7–11) years and a mean (SD) body mass index of 22.2 (4) kg/m^2^. All enrolled patients underwent a 3-month trial of medical treatment to assess their response (Figure 2); unfortunately, nine patients (9.9%) could not tolerate the drug therapy due to the development of drug-related side-effects, despite dose adjustment, and the significantly lower scores determined at time of shift to receive IDB injection after a mean (SD; range) duration of medical therapy of 48.7 (10.2; 35–65) days. During the 3-month medical treatment trial, 22 patients (24.2%) showed no or minimal improvement and refused to continue, so were shifted to receive IDB injection. In all, 60 patients (65.9%) responded to medical treatment; 48 patients showed progressive significant decrease in their subjective scores and continued their 12-month follow-up uneventfully with no or minimal side-effects, whilst 12 patients developed recurrent manifestations of OAB after a mean (SD; range) duration of 6 (1.1; 4–7) months and refused to repeat the trial, so shifted to receive IDB injection (Table 1). Collectively, the medical trial outcome included a 12-month TS rate of 52.7%, the recurrence rate was 13.2%, and the TF rate of 34.1%.

**Table 1.** Outcome of medical treatment of the studied patients with OAB.

			Failed medical therapy (*n* = 43)		
Time	Outcome data	Successful medical therapy (*n* = 48)	Intolerant to continue (*n* = 9)	Failure to respond (*n* = 22)	Recurrent OAB (*n* = 12)
Initiation of therapy	OABSS, mean (SD)	9.8 (1.1)	12.3 (1.6)	14.3 (0.9)	13.4 (1.1)
	PPBC score, mean (SD)	4.8 (0.9)	5.1 (0.6)	5.1 (0.8)	4.8 (0.6)
Termination of therapy	Time, mean (SD)	12 months	48.7 (10.2) days	3 months	6 (1.1) months
	OABSS, mean (SD)	4.8 (1.3)*	6.7 (1)*	13.4 (1.1)	12.4 (0.9)
	PPBC score, mean (SD)	2.1 (0.6)*	2.7 (0.9)*	5 (0.6)	4.5 (0.7)

*Significant vs time of initiation of therapy.

Patients who were refractory, intolerant or unresponsiveness to medical treatment (*n* = 43) underwent IDB uneventfully without intraoperative or postoperative complications. The mean (SD; range) operative time was 19 (3.6; 15–25) min and all patients were discharged once fully awake and ready to go home after a mean (SD; range) hospital stay of 2.9 (0.8; 2–4) h.

**Figure 3.**
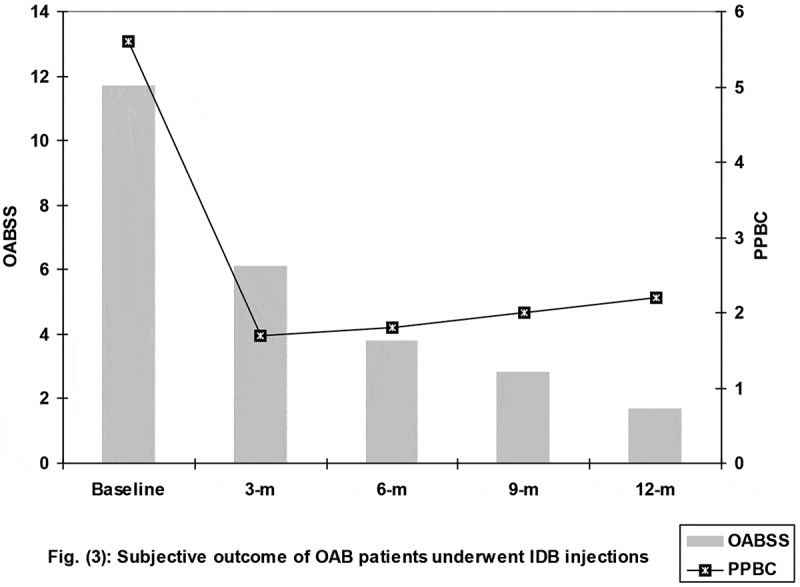
Subjective outcome of the patients with OAB who underwent IDB injections. m, months.

All patients showed progressively decreasing subjective scores with a significant difference compared to baseline scores (Figure 3). The MBC of the patients showed a significant increase at end of the 12-month follow-up compared to that determined at the start of treatment, despite being significantly lower than the EBC. Moreover, the calculated percentage of the capacity deficit was lower at end of follow-up compared to at the start of treatment. Furthermore, at the end of the follow-up, the voided volume and Q_max_ were significantly higher with significantly lower PVR in all patients compared to their measures at the start of treatment (Table 2).

**Table 2.** Objective evaluation of the outcomes of the studied patients with OAB.

Variable		At start of treatment, mean (SD)	At 12-month follow-up, mean (SD)
Bladder capacity	EBC, mL	315.6 (3)	
	MBC, mL	173.9 (41.4)*	257.9 (68.4)*†
	% of deficit	42.4 (15.4)	12.6 (27.2)†
Uroflowmetry	Voided volume, mL	117.4 (28)	168.7 (40.8)†
	Q_max_, mL/s	16.5 (6.1)	24 (4.8)†
	PVR, mL	25.2 (10.6)	12.2 (3.5)†

*Significant difference vs expected capacity; †significant difference vs time of initiation of therapy (baseline).

During the follow-up period, four patients who had IDB developed recurrent OAB manifestation after IDB injection for a TF rate of 9.3%. The mean (SD; range) duration of treatment efficacy was 11.7 (1.2; 5–12) months. Two patients who had recurrent manifestations found the result was good for ~10 months and so had no objection to repeating the trial of injection if it suspected to provide a similar outcome. Parents of the third patient had early recurrent manifestations at 155 days after IDB and found the trial was disappointing and refused to repeat the trial of injection. The parents of the fourth patient had recurrence 10 months after IDB injection and found it satisfactory but asked to repeat the trial of medical treatment for financial reasons.

As regards patients/parent satisfaction of the outcome, 30 patients/parents (69.7%) found that the outcome of IDB was very satisfactory, six (14%) found it satisfactory, six (14%) found it good, and only one (2.3%) found it disappointing and poor.

## Discussion

The present study included only patients who failed to respond to biofeedback therapy and received a 3-month trial of medical treatment with an anti-cholinergic drug; 22 patients failed to respond, nine were intolerant to treatment, and 12 developed recurrent OAB manifestations after initial success. These 43 patients (47.3%) were considered as medical TFs and underwent IDB injection.

These figures for the outcome of medical treatment mirror multiple previous studies and comparative trials. McDowell et al. [15] found anticholinergics were effective in 58.6% and 83.3% of males and females, but significant side-effects were experienced in 41.4% and 22.2% of males and females, respectively. Also, Quintiliano et al. [16], in a placebo-controlled study, evaluated the efficacy of parasacral TENS or oxybutynin for the management of OAB and reported complete resolution of symptoms in 46% vs 20%, respectively, and an oxybutynin discontinuation rate of 13% secondary to the development of side-effects that patients could not tolerate. Contrary to the reported 3-month success rate for medical treatment, Gleason et al. [17] reported an overall good symptom response of 97% with a transdermal oxybutynin patch, but a side-effect rate of 35% causing discontinuation in 20%.

During the follow-up after IDB injection, only four patients had recurrent OAB manifestations for a TF rate of 9.3% and 36 patients/or parents were satisfied to very satisfied by the IDB result for a satisfaction rate of 83.7%. The reported TS and satisfaction rates by the outcome of IDB were significantly higher in comparison to those of medical treatment. Similarly, McDowell et al. [15] reported, in patients where anti-cholinergic drugs were ineffective, complete success after IDB in 74.2% and 54.5% of males and females, respectively; partial success in 20% and 18.2% of males and females; and TF was significantly higher in females (22.7%) than males (2.9%). Thereafter, Khan et al. [18], using IDB injection of 300 U in children who had anti-cholinergic refractory OAB, reported improved continence after the initial IDB in 54%, with cystometric capacity increasing by 46% and maximum detrusor pressure decreasing by 43% after initial IDB.

Consistent with the reported 12-month success rates and effectiveness of IDB injection, Ladi-Seyedian et al. [19] reported 1-, 2-, 3-, 5- and 6-year success rates of 75%, 45.5%, 37.5%, 33% and 29.1% in patients with UI due to neuropathic detrusor overactivity secondary to myelomeningocoele by the use of intravesical electromotive botulinum toxin type A administration. Whilst, Lane et al. [20] documented that non-surgical protocol for patients with neurogenic bladders and detrusor leak-point pressures >40 cmH_2_O, including IDB injection in conjunction with anticholinergics is safe and effective, with a 3-year success rate of 85%

In the present study, all patients received IDB injection of 100 U and the procedure resulted in no postoperative problems and this could be attributed to the small injected dose that minimised drug-induced side-effects without compromising the outcome. Also, the injection technique spared the trigone and sphincter to avoid postoperative outflow obstruction, and lastly, proper preoperative eradication of UTI and use of postoperative urinary antiseptics and broad-spectrum antibiotics prevented the possibility of postoperative infectious complications.

These findings coincide with Lahdes-Vasama et al. [21] who found IDB, in a dose of 50–100 U injected at 15–20 detrusor sites, effectively reduced day-time wetting, significantly increased bladder volume and decreased detrusor overactivity in children with urge UI refractory to anti-cholinergics. Also, Santos et al. [22] documented that in patients with OAB/idiopathic detrusor overactivity, 100  U onabotulinumtoxinA injected in 20 sites above the trigone markedly decreased UI, frequency and urgency episodes and improved quality of life. Thereafter, Léon et al. [23] reported improvements without any complaints during bladder voiding for 100% of their series of children with non-neurogenic OAB and found OAB disappeared completely after one injection in 67% and repeated injections in 33%, with improved compliance early-on in 50% and at 1-year in 100% of patients. Also, ‘t Hoen et al. [24] reported a success rate of 87.5% with onabotulinumtoxinA 100 U injection in children with therapy-refractory dysfunctional voiding after a median follow-up of 13 months, and found all of these children were no longer daily incontinent and 65.3% of patients became completely dry.

In support of the efficacy of IDB injection as a line of management for OAB cases refractory or intolerant to anti-cholinergic therapy, irrespective of its brand, Bottet et al. [25] reported a 2-year success rate of 87% in cases refractory to IDB injection on switching to a different brand of botulinum toxin A (Dysport®).

## Conclusion

For children with OAB refractory or resistant to biofeedback therapy, anti-cholinergic drugs must be tried first and IDB should be reserved for cases who fail to respond, are intolerant or have recurrence after medical treatment. IDB using 100 U Botox at 20 injection sites, with trigone and sphincter sparing, is a successful modality with a high satisfaction rate and free of postoperative problems.
